# Treatment of Acne

**Published:** 1879-09

**Authors:** 


### TREATMENT OF ACNE.

The treatment of acne punctata, with glycerine and sulphur, as first proposed by Mr. Erasmus Wilson, is endorsed by Dr.

J. G. Parsons, in the *British Medical Journal.* He thinks, however, that a far more efficacious way of applying the sulphur, is to dust it upon the face every night, using an ordinary puff. The sulphur may be perfumed with a drop or two of Otto Rose. This will often effect a cure in one or two weeks.

